# Supplementary material for: A Mycobacterium tuberculosis-specific subunit vaccine that provides synergistic immunity upon co-administration with Bacillus Calmette-Guérin
Source: Nat Commun. 2021 Nov 18;12:6658. doi: 10.1038/s41467-021-26934-0 (PMC8602668; doi:10.1038/s41467-021-26934-0)
Supplement: Supplementary file 1 — Supplementary Information [file 41467_2021_26934_MOESM1_ESM.pdf]

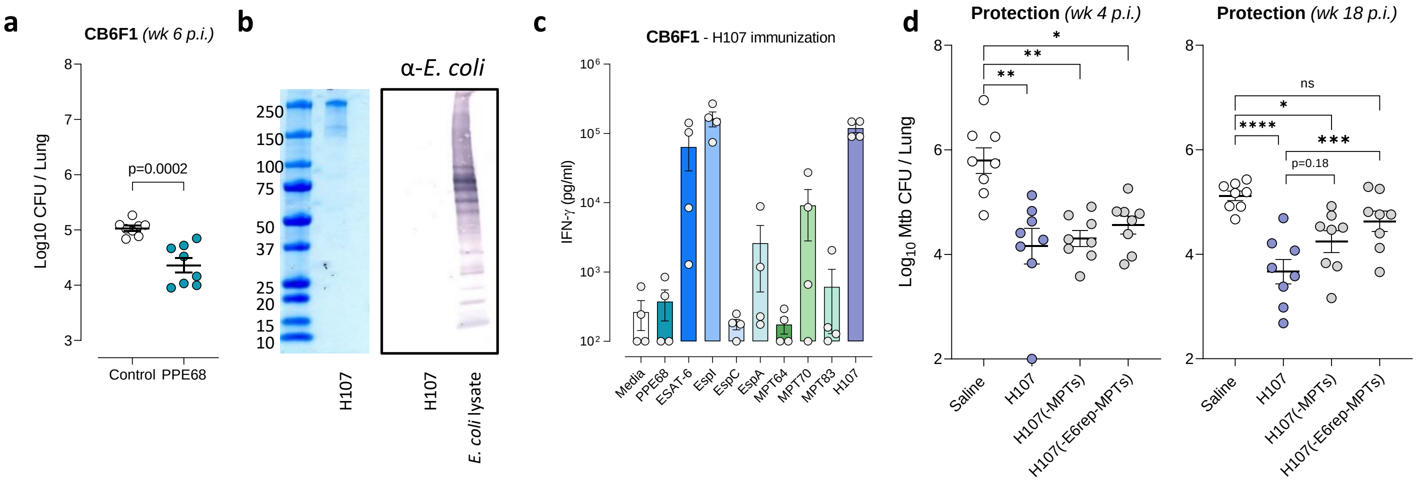

Fig. S1. Vaccine protection of PPE68 and antigen responses after H107 immunization.

(a) Bacterial numbers were determined in the lungs of control (non-vaccinated) and PPE68-vaccinated mice six weeks post aerosol Mtb challenge (n=8). Two-tailed unpaired t-test. **(b)** Coomassie stained SDS-PAGE gel shows purified H107 (224 kDa). Western blot analysis with anti-*E. coli* antibody detected no residual *E. coli* contaminants in the purified H107. **(c)** CB6F1 mice were vaccinated with H107 three times s.c. and splenocytes were harvested two weeks after the third vaccination. Splenocytes were restimulated *ex vivo* with medium, individual recombinant antigens, or recombinant H107 for three days. The levels of IFN-γ (pg/mL) were measured in the culture supernatant (n=4). Symbols indicate individual mice. Box plots indicate median, interquartile range, and minimum and maximum values. **(d)** CB6F1 mice were immunized three times s.c. with saline or CAF®01 formulated with 1 µg H107, H107(-MPTs) (lacking MPT64, MPT70 and MPT83) or H107(-E6rep-MPTs) (lacking ESAT-6 repeats and MPT64, MPT70 and MPT83). The bacterial burden was determined in the lungs 4 and 18 weeks post infection (p.i.) (n=8). Symbols indicate individual mice with mean±SEM. p-values; *p<0.05, **p<0.01, , ***p<0.001 ****p<0.0001, and ns (non-significant) based on one-way ANOVA with Tukey's post-test. Exact p-values: wk 4 p.i. Saline vs H107 p=0.0001, saline vs. H107(-MPTs) p=0.0004, saline vs. H107(-E6rep-MPTs) p=0.0043; wk 18 p.i. Saline vs H107 p<0.0001, saline vs. H107(-MPTs) p=0.0116, H107 vs. H107(-E6rep-MPTs) p=0.0002.

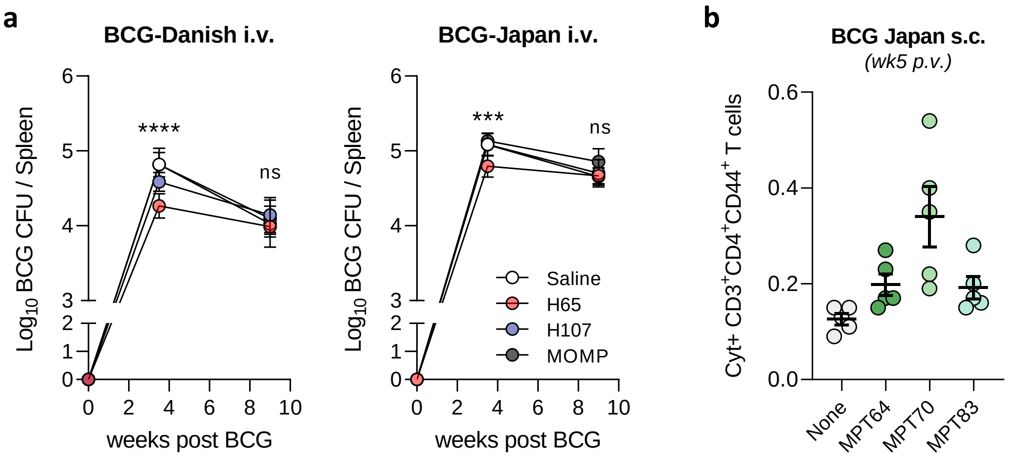

Fig. S2. Cross reactivity of H65 and H107 with BCG-Danish and BCG-Japan

(a) Inhibition of BCG-Danish and BCG-Japan colonization in the spleen. CB6F1 mice were immunized with H65/CAF®01, H107/CAF®01, or MOMP/CAF®01 and then injected with BCG intravenously (i.v.) six weeks after the last immunization. BCG CFUs were enumerated in the spleen 3.5 and 9 weeks post BCG inoculation (n=8; symbol, mean±SEM). One-way ANOVA with Tukey's Multiple Comparison test. p-values; Saline vs. H65 shown, ***p<0.0001 [p=0.0009], ****p<0.0001 and ns (non-significant). **(b)** MPT70-, MPT83-, and MPT64-specific immune responses induced by BCG-Japan. CB6F1 mice were vaccinated with BCG-Japan s.c. and the immune responses were assessed five weeks post vaccination (n=4; line, mean±SEM).

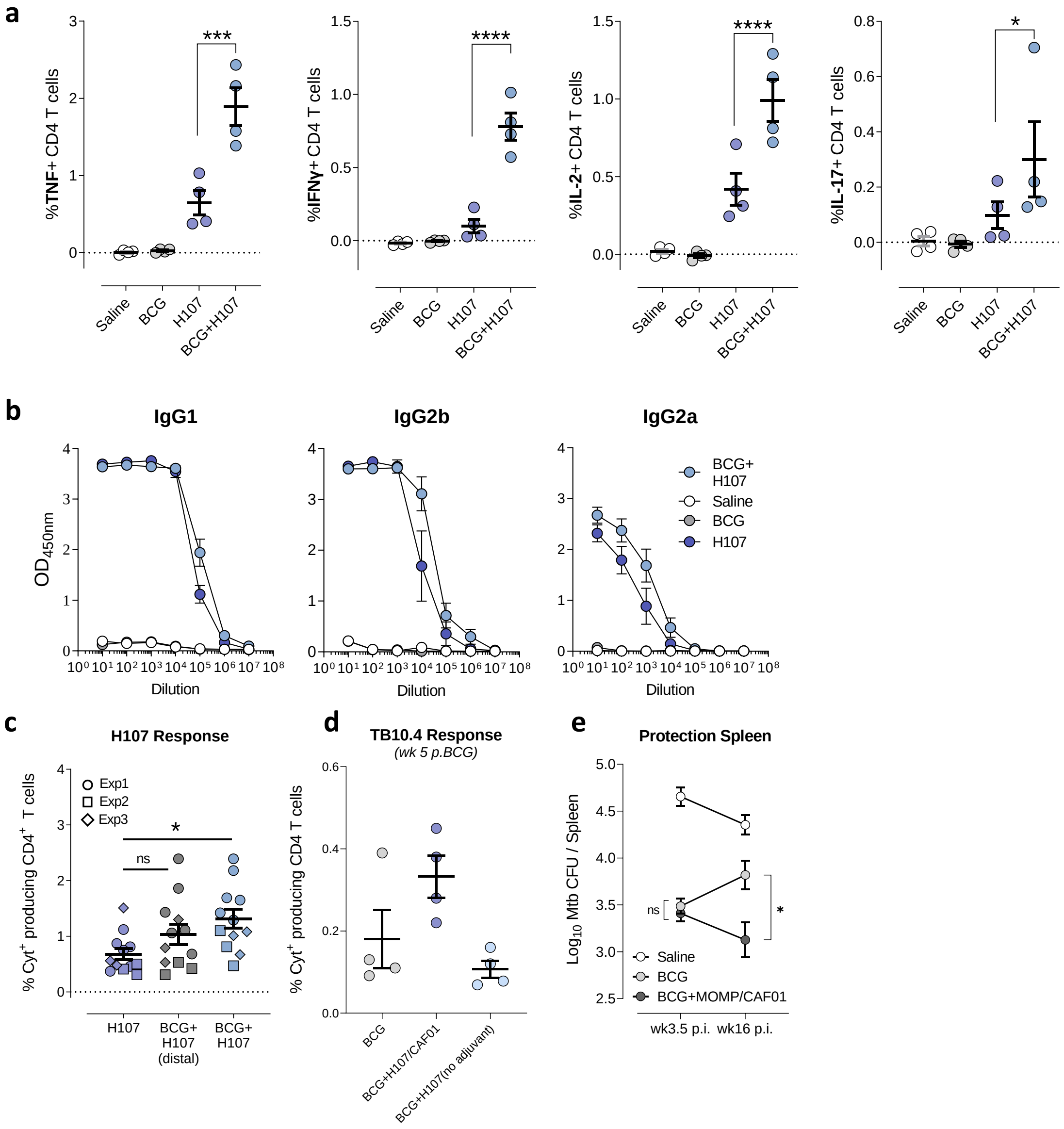

Fig. S3. H107- and BCG-specific immune responses after co-administration

(a,b) CB6F1 mice were vaccinated with saline, BCG-Danish, 1µg H107/CAF®01, or a co-administration regimen of BCG+H107. One week after final H107 immunization, **(a)** the percent of Spleen CD4 T cells producing cytokines TNF, IFNγ, IL-2, and IL-17 was determined by ICS, and **(b)** H107-specific antibodies (IgG1, IgG2b, and IgG2a) measured in blood plasma by ELISA, (n=4). **(c)** Comparison of H107-specific CD4 T cells after BCG+H107 were co-administered such as to drain to the same lymph node or different (distal) lymph nodes. The percentage of total cytokine-producing (IFN-γ, IL-2, TNF and/or IL-17A via Boolean OR gating) CD44^{high} CD4 T cells after *ex vivo* restimulation of splenocytes with H107 protein one week post final H107 vaccination are shown. Data compiled from three independent experiments, as indicated. (H107 vs BCG+H107 p=0.0177) **(d)** CB6F1 mice were vaccinated with BCG-Danish or a co-administration regimen of BCG+H107 with or without adjuvant CAF®01. Five weeks after BCG (one week after the final H107 vaccination), the percentage of total cytokine-producing CD44^{high} CD4 T cells after *ex vivo* restimulation of splenocytes with TB10.4 protein (n=4) was determined. **(e)** CB6F1 mice were vaccinated s.c. with saline, BCG-Danish, or BCG+ 1µg MOMP/CAF®01 followed by two MOMP/CAF®01 boosts. Mice were rested for 6 weeks and then challenged with aerosolized Mtb Erdman (n=7-8). The bacterial burden was accessed in the spleen 3.5 and 16 weeks post Mtb infection (p.i.). . Data plotted as average mean ± SEM (line) of individual mice (symbols in a,c,d). One-Way ANOVA with Tukey's multiple comparisons test. p-values; *p<0.05 [(a) p=0.499), (c) p=0.0177, (d) p=0.0104), ***p<0.001 [(a) p=0.0003], ****p<0.0001, and ns (non-significant).

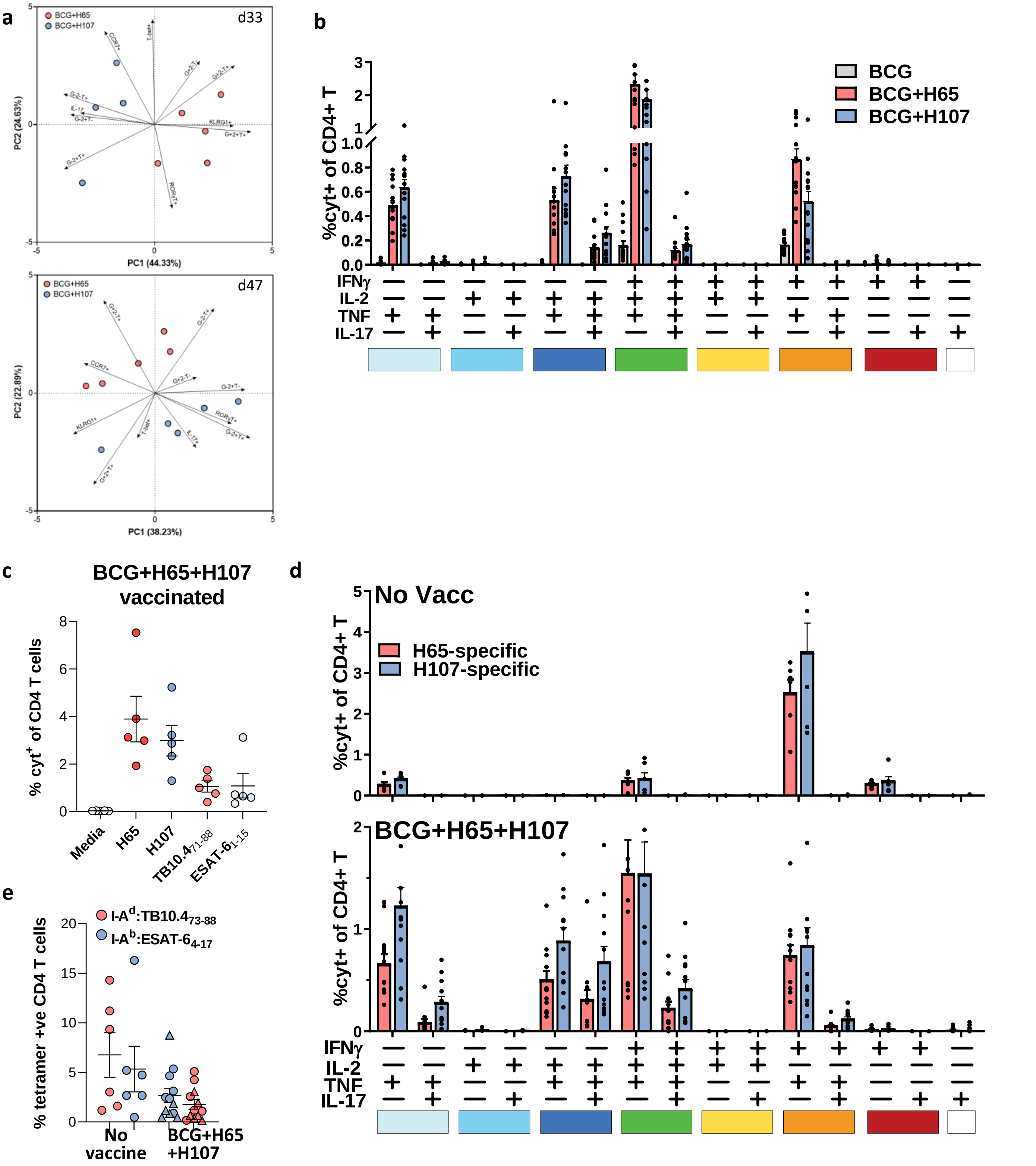

Fig. S4. Characterization of immune responses after BCG co-administered with H65 and/or H107 before and after Mtb challenge . (a,b) CB6F1 mice were immunized s.c. with either BCG, BCG+H65/CAF®01 or BCG+H107/CAF®01. Five to seven weeks post final immunization, splenocytes were restimulated *ex vivo* with either H65 (BCG, BCG+H65) or H107 (BCG+H107) for intracellular cytokine staining (ICS). **(a)** Principal component analysis (PCA) of vaccine-specific CD4 T cells from assessed five (top) and seven (bottom) weeks after immunization for TNF/ IFN-γ/IL-2, IL-17, RORγT, T-bet, KLRG1, and CCR7 expression. Percentages on axes indicate variance explained by each PC. **(b)** Frequency of antigen-specific splenic CD4 T cells producing combinations of IFN-γ, IL-2, TNF, and/or IL-17 from BCG (grey) BCG+H65 (red) and BCG+H107 (blue) mice, five to seven weeks after immunization (BCG, BCG+H65 n=15, BCG+H107 n=14; bars, mean±SEM). **(c-e)** CB6F1 mice were immunized s.c. with saline or a co-administration of BCG+ simultaneous H65+H107/CAF®01. **(c)** Frequency of antigen-specific splenic CD4 T cells assessed six weeks after BCG+H65+H107/CAF®01 immunization by *ex vivo* stimulation with H65, H107, or single peptide epitopes TB10.$4_{71\text{-}88}$ or ESAT-.$6_{1\text{-}15}$ for cytokine expression (TNF,IFN-γ,IL-2 and/or IL-17A) by ICS (n=5). (d,**e)** Frequency of antigen-specific lung CD4 T cells producing combinations of IFN-γ, IL-2, TNF, and/or IL-17 after *ex vivo* stimulation with H65 (red) or H107 (blue), from control (top, n=6) and BCG+H65+H107-vaccinated (bottom, n=12) mice 27-33 days after aerosol Mtb challenge (bars, mean±SEM). **(e)** The percentage of I-A^d:TB10.$4_{73\text{-}88}$ (red) or I-A^b:ESAT-$6_{4\text{-}17}$ (blue) tetramer binding $CD44^{high}$ CD4 T cells identified in the lungs of non-vaccinated and BCG+H65+H107-vaccinated mice 27 (triangles, n=6) and 33 (circles, n=6) days after aerosol Mtb infection (No Vacc. n=6, BCG+H65+H107 n=12). Symbols indicate individual animals and lines indicate mean±SEM.

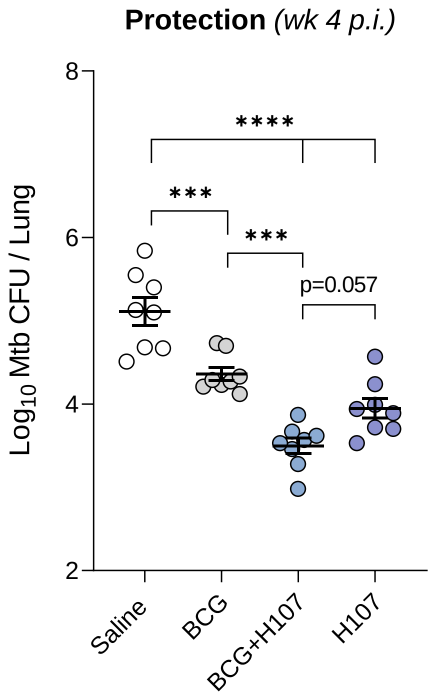

Fig S5. Lung bacterial load in vaccinated and Mtb infected mice

CB6F1 mice were immunized once s.c. with 0.5×10^6 CFU BCG Danish, three times s.c. with saline or 1 µg H107/CAF®01, or co-administered BCG+H107/CAF®01 as described previously (n=8). Six weeks later mice were infected with aerosol Mtb Erdman with a dose corresponding to 25-50 CFU per mouse. The bacterial burden was determined in the lungs four weeks post infection (p.i.). Individual values are shown, line indicates mean±SEM. One-Way ANOVA with Tukey's multiple comparisons test. p-values; ***p<0.001 [Saline vs BCG p=0.0007, BCG vs BCG+H107 p=0.0001], ****p<0.0001.

Table S1: Antigen expression of BCG substrains

	Strain	PPE68 (Rv3873)	EsxA (Rv3675)	EspI (Rv3876)	EspC (Rv3615c)	EspA (Rv3615c)	MPT64 (Rv1980c)	MPT70 (Rv2875)	MPT83 (Rv2873)
	M.tuberculosis	+++	+++	+++	+++	+++	+++	+++	+++
	M.bovis	+++	+++	+++	+++	+++	+++	+++	+++
Early BCG strains (lack RD1)#	BCG Russia	-	-	-	(+++)	(+++)	+++	+++	+++
	BCG Japan	-	-	-	(+++)	(+++)	+++	+++	+++
	BCG Moreau	-	-	-	(+++)	(+++)	+++	+++	+++
	BCG Sweden	-	-	-	(+++)	(+++)	+++	+++	+++
	BCG Birkhaug	-	-	-	(+++)	(+++)	+++	+++	+++
Modern BCG strains (Lack RD1 and RD2)#$	BCG Tice	-	-	-	(+++)	(+++)	-	(+)	(+)
	BCG Frappier	-	-	-	(+++)	(+++)	-	(+)	(+)
	BCG Pasteur	-	-	-	(+++)	(+++)	-	(+)	(+)
	BCG Danish	-	-	-	(+++)	(+++)	-	(+)	(+)
	BCG Glaxo	-	-	-	(+++)	(+++)	-	(+)	(+)
	BCG Prague	-	-	-	(+++)	(+++)	-	(+)	(+)
	BCG China	-	-	-	(+++)	(+++)	-	(+)	(+)

The RD1 region was lost during the attenuation of *M. bovis* (1908-21)
$ BCG substrains acquired after 1927 also the RD2 region and have a mutation in *sigK*

- gene not present on chromosome
+++ high protein expression
(+) low protein expression due to *sigK* mutation
(+++) high expression but the protein is not secreted due to a defect in the ESX-1 secretion system

Adapted from: Human Vaccines, 2009, 5, 70-78

Table S2: Overview of antigen modifications in H107

Name	Rv. No.	Protein length	Part included in H107	Modifications
PPE68	Rv3873	368	155-219, GGS linker, 242-305	Regions with homology to BCG removed (Okkels et. al. 2003)
ESAT-6 / EsxA	Rv3875	95	2-95	No modifications
EspI	Rv3876	666	2-666	K425Q (inactivation of ATP binding site) (Chen et. al., 2016)<br>C537S, C590S (cysteine's replaced with serine's)
EspC	Rv3615c	103	2-54	C-terminal part removed for compatibility with ESAT-6 free IGRA (Ruhwald et. al., 2017)
EspA	Rv3616c	392	2-131, 155-392	Transmembrane / hydrophobic amino acids removed
MPT64	Rv1980c	228	24-228	Signal sequence omitted<br>C29S, C41S (cysteine's replaced with serine's)
MPT70	Rv2875	193	31-193	Signal sequence omitted<br>C38S, C172S (cysteine's replaced with serine's)
MPT83	Rv2873	220	31-220	Signal sequence omitted<br>C64S, C198S (cysteine's replaced with serine's)
