# Supplementary material for: A Mycobacterium tuberculosis-specific subunit vaccine that provides synergistic immunity upon co-administration with Bacillus Calmette-Guérin
Source: Nat Commun. 2021 Nov 18;12:6658. doi: 10.1038/s41467-021-26934-0 (PMC8602668; doi:10.1038/s41467-021-26934-0)
Supplement: Supplementary file 3 — Reporting Summary [file 41467_2021_26934_MOESM3_ESM.pdf]

nature portfolio

Corresponding author(s): Rasmus Mortensen

Last updated by author(s): Sep 30, 2021

Reporting Summary

Nature Portfolio wishes to improve the reproducibility of the work that we publish. This form provides structure for consistency and transparency in reporting. For further information on Nature Portfolio policies, see our Editorial Policies and the Editorial Policy Checklist.

Statistics

For all statistical analyses, confirm that the following items are present in the figure legend, table legend, main text, or Methods section.

n/a	Confirmed	
☐	☒	The exact sample size (*n*) for each experimental group/condition, given as a discrete number and unit of measurement
☐	☒	A statement on whether measurements were taken from distinct samples or whether the same sample was measured repeatedly
☐	☒	The statistical test(s) used AND whether they are one- or two-sided<br>*Only common tests should be described solely by name; describe more complex techniques in the Methods section.*
☒	☐	A description of all covariates tested
☐	☒	A description of any assumptions or corrections, such as tests of normality and adjustment for multiple comparisons
☐	☒	A full description of the statistical parameters including central tendency (e.g. means) or other basic estimates (e.g. regression coefficient) AND variation (e.g. standard deviation) or associated estimates of uncertainty (e.g. confidence intervals)
☐	☒	For null hypothesis testing, the test statistic (e.g. *F*, *t*, *r*) with confidence intervals, effect sizes, degrees of freedom and *P* value noted<br>*Give P values as exact values whenever suitable.*
☒	☐	For Bayesian analysis, information on the choice of priors and Markov chain Monte Carlo settings
☒	☐	For hierarchical and complex designs, identification of the appropriate level for tests and full reporting of outcomes
☒	☐	Estimates of effect sizes (e.g. Cohen's *d*, Pearson's *r*), indicating how they were calculated

Our web collection on statistics for biologists contains articles on many of the points above.

Software and code

Policy information about availability of computer code

Data collection	No software was used for data collection.
Data analysis	FlowJo v10 (Treestar) for Flow cytometry and Prism v.8/9 (GraphPad) for statisical analyses.

For manuscripts utilizing custom algorithms or software that are central to the research but not yet described in published literature, software must be made available to editors and reviewers. We strongly encourage code deposition in a community repository (e.g. GitHub). See the Nature Portfolio guidelines for submitting code & software for further information.

Data

Policy information about availability of data

All manuscripts must include a data availability statement. This statement should provide the following information, where applicable:

- Accession codes, unique identifiers, or web links for publicly available datasets
- A description of any restrictions on data availability
- For clinical datasets or third party data, please ensure that the statement adheres to our policy

The data that support the findings of this study are available from the corresponding author upon request. There are no restrictions on data availability. The data reported here are provided in the Source Data file provided with this paper.

Field-specific reporting

Please select the one below that is the best fit for your research. If you are not sure, read the appropriate sections before making your selection.

☒ Life sciences ☐ Behavioural & social sciences ☐ Ecological, evolutionary & environmental sciences

For a reference copy of the document with all sections, see nature.com/documents/nr-reporting-summary-flat.pdf

Life sciences study design

All studies must disclose on these points even when the disclosure is negative.

Sample size

In our standardized murine aerosol Mtb challenge model, we powered the experiments for detecting a treatment effect (protection) of 0.5 log10 CFU reduction in lungs compared to non-treated controls with a type I error rate of 5% (α=0.05), a power of 80% and standard deviation of 0.35 log10 CFU (based on previous experiments). This results in n=8 mice per group for primary analysis.
For comparative T cell magnitudes and phenotypical analysis, 4-6 mice per group were used based on our previous studies (e.g. PMIDs: 27554293, 33879592) comparing T cell responses in immunized and Mtb-infected mice for these and similar parameters. A minimum of 4 mice per condition was used determine, being increased to improved statistical robustness up to 6 mice as feasible given the number of animals to compare at a single time point under a single analysis. Experimental design that allowed for paired analysis (such as in Fig. 4) was included, as possible, to maximize statistical power within such sample sizes.
For the study with human samples, no sample size calculation was performed, but based on previous studies (PMIDs 27409590, 32499216, 29602771) from our group we expect a difference in magnitude of response when comparing MTB300 reactivity in QFT+ vs. QFT- individuals. The final sample size used was based on the samples available from the clinical site.

Data exclusions

No data was excluded from the analyses

Replication

All experimental replications were successful and supported the data presented.
Among the studies performed once:
The TCR sequence analysis (performed by ENPICOM) was included as an exploratory approach to increase the understanding of the vaccine-induced T cell imprint. These analyses were time and resource intensive and since the data were consistent with the flow cytometry analysis, we did not find a repeat experiment necessary. The human participant study was based on the statistical significance of the results acquired and the samples available from the clinical site, and the long-term Mtb infection time point was due to the consistency with the shorter term data, the statistical significance of the results, ethical considerations, and time to publication reporting considerations.

Randomization

Mice were randomly assigned to cages and treatment upon arrival to our animal facility. For each time point in the study, mice were randomly selected for primary analysis.
Human participants were allocated into experimental groups based on IGRA status depicting their previous Mtb exposure (QFT+/-) as that was the intended comparison of the study.

Blinding

The investigator was not involved in CFU data collection. Organ homogenization, plating, and CFU counting was performed by an experienced technician, who was not involved in study design and/or data analysis. The investigator was not specifically blinded during analysis and interpretation, however the statistical comparisons to be performed between groups (ANOVA with Tukey's posttest) was pre-determined, and therefore was not impacted.
Similarly, the sample preparation and data acquisition of T cell immunological data was performed by an effectively-blinded technician who was not involved in the study design. The investigator applied uniform gating strategies, etc to all samples being compared. However, as analysis of the T cells responses would inherently reveal the vaccine status/history of the animals, blinding of the investigator was not realistically possible for the studies performed.
TCR sequence analysis was performed by a third party entity (ENPICOM), who performed statistical comparisons without scientific investment in the outcome or conclusions.
Individuals performing the experiments and collecting the raw data from the human participants were blinded to cohort assignment QFT+ vs QFT-. Participants from both groups were included in each experiment. Individuals analyzing the data were not blinded since the comparisons were dependent on knowing the respective cohort.

Reporting for specific materials, systems and methods

We require information from authors about some types of materials, experimental systems and methods used in many studies. Here, indicate whether each material, system or method listed is relevant to your study. If you are not sure if a list item applies to your research, read the appropriate section before selecting a response.

Materials & experimental systems

n/a	Involved in the study
☐	☒ Antibodies
☒	☐ Eukaryotic cell lines
☒	☐ Palaeontology and archaeology
☐	☒ Animals and other organisms
☐	☒ Human research participants
☒	☐ Clinical data
☒	☐ Dual use research of concern

Methods

n/a	Involved in the study
☒	☐ ChIP-seq
☐	☒ Flow cytometry
☒	☐ MRI-based neuroimaging

Antibodies

Antibodies used

CD3-BV650 (Biolegend, clone: 17A2, catalog #100229,lot #B282105)
CD3-BV605 (BD biosciences, clone: 145-2C11, catalog #563004,lot #1111157)
CD4-BV510 (Biolegend, clone: RM4.5, catalog #100559,lot #B294301)
CD4-FITC (BD biosciences, clone: RM4.5, catalog #553047,lot #6110899)
CD4-BV650 (BD biosciences, clone: GK1.5, catalog #563232,lot #4352667)
CD8-BV421 (Biolegend, clone: 53-6.7, catalog #100738,lot #B308976)
CD19-BV510 (Biolegend, clone: 6D5, catalog #115545,lot #B307447)
CD19-PerCP-Cy5.5 (BD biosciences, clone: 1D3, catalog #551001,lot #8116599)
CD44-Alx700 (Biolegend, clone: IM7, catalog #103026,lot #B340054)
KLRG1-BV711 (BD biosciences, clone: 2F1, catalog #564014,lot #1120802)
CCR7-APC/e780 (ThermoFisher, clone: 4B12, catalog #47-1971-82,lot #4281672)
CCR7-PE/Cy7 (eBioscience, clone: 4B12, catalog #25-1971-82,lot #2191978)
CD62L-FITC (eBioscience, clone: MEL-14, catalog #553150,lot #9086503)
CXCR3-BV421 (Biolegend, clone: CXCR3-173, catalog #126529,lot #B252940)
CXCR3-PerCP/Cy5.5 (ThermoFisher, clone: CXCR3-173, catalog #45-1831-82,lot #1994230)
IFNγ-PE-Cy7 (eBioscience, clone: XMG1.2, catalog #25-7311-82,lot #2254266)
IFNγ-BV421 (BD biosciences, clone: XMG1.2, catalog #563376,lot #8144795)
TNF-PE (eBioscience, clone: MP6-XT22, catalog #12-7321-82,lot #2024856)
IL-2-APC/Cy7 (BD biosciences, clone: JES6-5H4, catalog #560547,lot #1083066)
IL-17A-PerCP/Cy5.5 (eBioscience, clone: eBio17B7, catalog #45-7177-82,lot #2082373)
Tbet-eflour-660 (eBioscience, clone: eBio-4B10, catalog #eBio 50-5825-82,lot #2239941)
RORγT-PE/CF594 (BD biosciences, clone: Q31-378, catalog #562684,lot #1124919)
CD16/CD32-purified (BD biosciences, clone: 2.4G2, catalog #553142,lot #0148675)
CD28-purified (BD biosciences, clone: 37.51, catalog #553295,lot #0357876)
CD49d-purified (BD biosciences, clone: 9C10-MFR4.B, catalog #553313,lot #1138960)
IFNγ-purified (BD Pharmingen, clone: R4-6A2, catalog #551216,lot #336058)
IFNγ-biotin (BD Pharmingen, clone: XMG1.2, catalog #554410,lot #9176274)

Validation

Validation of all primary commercial antibodies for the species and application was warranted by the vendors. Validation statement can be found on the manufacturers' website.

Animals and other organisms

Policy information about studies involving animals; ARRIVE guidelines recommended for reporting animal research

Laboratory animals

Six-to-eight week old female B6C3F1 (H2b,k) and CB6F1 (H2b,d) mice were obtained from Envigo (Netherlands). Mice were randomly assigned to cages of eight upon arrival. Before the initiation of experiments, mice had at least one week of acclimatization in the animal facility. During the course of the experiment, mice had access to irradiated Teklad Global 16% Protein Rodent Diet (Envigo, 2916C) and water ad libitum. Mice were housed at an ambient temperature of 20-23°C and 45-65% relative humidity on a 12hr/12hr light/dark cycle with 15 minutes dusk and dawn transition periods under Biosafety Level (BSL) II or III conditions in individually Type III ventilated cages (Scanbur, Denmark) and had access to nesting material (enviro-dri and soft paper wool; Brogaarden) as well as enrichment (aspen bricks, paper house, corn, seeds, and nuts; Brogaarden).

Wild animals

No wild animals were used in the study.

Field-collected samples

No field collected samples were used in the study.

Ethics oversight

Experiments were conducted in accordance with the regulations set forward by the Danish Ministry of Justice and animal protection committees by Danish Animal Experiments Inspectorate Permits 2014-15-2934-01065 and 2019-15-0201-00309 in compliance with European Community Directive 2010/63/EU of the European parliament and of the council of 22 September 2010 on the protection of animals used for scientific purposes, as well as Directive 86/609 and the U.S. Association for Laboratory Animal Care recommendations for the care and use of laboratory animals. The experiments were approved by the local animal protection committee at Statens Serum Institut, IACUC, headed by DVM Kristin Engelhart Illigen.

Note that full information on the approval of the study protocol must also be provided in the manuscript.

Human research participants

Policy information about [studies involving human research participants]

Population characteristics	We enrolled 22 QFT+ (QuantiFERON Gold In-Tube, Cellestis) and 10 QFT- individuals at the Antiviral Research Center Clinic, University of California San Diego (UCSD). Subjects did not have any clinical or radiographic signs of active TB. None of the study subjects endorsed vaccination with BCG, or had laboratory evidence of HIV or Hepatitis B. QFT+ subjects were between 18-61 years old (median 39) and 55% were male, and QFT- were between 34-60 (median 46) with 80% male.
Recruitment	Participants were recruited on the basis of their history of a tuberculin skin test (TST) through flyers at the Antiviral Research Center Clinic, UCSD. Mtb infection was determined by QuantiFERON. A physical exam and/or chest X-ray ruled out active tuberculosis.
Ethics oversight	All participants provided written informed consent to the study and ethical approval to carry out the work is maintained through the La Jolla Institute for Immunology Institutional Review Board.

Note that full information on the approval of the study protocol must also be provided in the manuscript.

Flow Cytometry

Plots

Confirm that:

- [x] The axis labels state the marker and fluorochrome used (e.g. CD4-FITC).
- [x] The axis scales are clearly visible. Include numbers along axes only for bottom left plot of group (a 'group' is an analysis of identical markers).
- [x] All plots are contour plots with outliers or pseudocolor plots.
- [x] A numerical value for number of cells or percentage (with statistics) is provided.

Methodology

Sample preparation	Single cell suspensions from tissue samples were prepared by mechanically forcing the tissue through a 100 um cell strainer. For lung samples, a pre-processing step was necessary in which lungs were ground using Auto-MACS C-tubes (Miltenyi Biotec) and treated with collagenase IV for 30-60 minutes at 37 degrees, 5% CO2. Cells were washed twice in RPMI and then resuspended in RPMI + 10% FCS. Cells were at this point on single cell level and ready for staining analysis.
Instrument	Samples were analyzed with a BD LSRFortessa™ using a BD™ High Throughput Sampler (HTS).
Software	Data management was carried out with BD FACSDiva™ v6.2 software. Data was analyzed using FlowJo software v.10 (Tree Star, Ashland, OR, USA).
Cell population abundance	No cell sorting was performed with FACS.
Gating strategy	For T-cell analyses, cells were gating for singlets (FSC-A/FSC-H), lymphocytes (FSC-A/SSC-A) and a CD4 T cell gate (CD3+ [BV650 or BV605] , CD4+ [BV510 or FITC]). Subsequently, CD44hi [AF-700] antigen specific cells were either gated by tetramer staining (MCH II tetramer [PE or BV421]) specific T cells, or cytokine producing T cells (IFNg [PE/Cy7], IL-2 [APC/Cy7], TNF [PE], IL-17 [PerCP/Cy5.5] or IFNg [BV421, IL-2 [APC/Cy7], TNF [PE], IL-17 [PerCP/Cy5.5]). An 'any' cytokine producing cell gatewas created via Boolean 'OR' gating in FlowJo.<br>Antigen-specific T cells were further characterized for their expression of T-bet [e660], RORgT [PE-CF594], CCR7 [PE/Cy7], and KLRG1 [BV-711].<br>Fixable Viability dye (viability-ef780 and ef506) was used to discriminate dead cells for all tetramer stainings and for ICS when possible.<br><br>Sample Gating for antigen specific CD4 T cells is shown in the figures.

- [x] Tick this box to confirm that a figure exemplifying the gating strategy is provided in the Supplementary Information.
